# Phosphorus recovery from wastewater and bio-based waste: an overview

**DOI:** 10.1080/21655979.2022.2077894

**Published:** 2022-06-05

**Authors:** Anna Witek-Krowiak, Katarzyna Gorazda, Daniel Szopa, Krzysztof Trzaska, Konstantinos Moustakas, Katarzyna Chojnacka

**Affiliations:** a Department of Advanced Material Technologies, Faculty of Chemistry, Wrocław University of Science and Technology, Wrocław, Poland; b Faculty of Chemical Engineering and Technology, Cracow University of Technology, Cracow, Poland; c School of Chemical Engineering, National Technical University of Athens, Athens, Greece

**Keywords:** Phosphorus, sewage sludge, valorization, waste management, wastewater

## Abstract

Phosphorus is one of the most important macronutrients needed for the growth of plants. The fertilizer production market uses 80% of natural, non-renewable phosphorus resources in the form of phosphate rock. The depletion of those deposits forces a search for other alternatives, including biological waste. This review aims to indicate the most important ways to recover phosphorus from biowaste, with particular emphasis on wastewater, sewage sludge, manure, slaughter or food waste. A comparison of utilized methods and directions for future research based on the latest research is presented. Combining biological, chemical, and physical methods with thermal treatment appears to be the most effective way for the treatment of wastewater sludge in terms of phosphorus recovery. Hydrothermal, thermochemical, and adsorption on thermally treated adsorbents are characterized by a high phosphorus recovery rate (over 95%). For animal by-products and other biological waste, chemical methods seems to be the most optimal solution with a recovery rate over 96%. Due to its large volume and relatively low phosphorus content, wastewater is a resource that requires additional treatment to recover the highest possible amount of phosphorus. Pretreatment of wastewater with combined methods seems to be a possible way to improve phosphorus recovery. A compressive evaluation of combined methods is crucial for future research in this area.

## GRAPHICAL ABSTRACT

**Figure:**
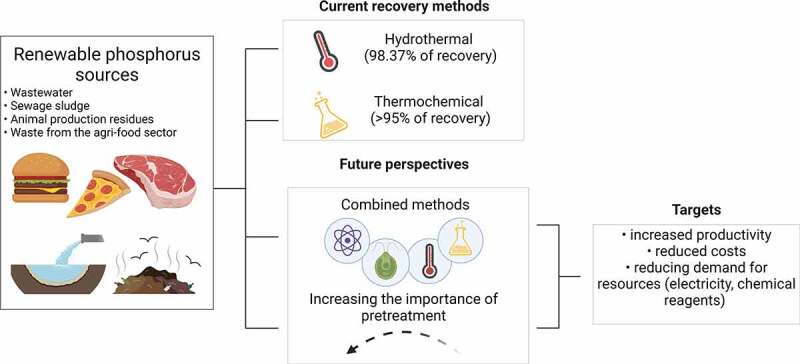

## Highlights

- The new, major manageable sources of phosphorus have been identified.
- The potential methods for phosphorus recovery and their advantages were compared.
- Advanced methods have been selected to improve the phosphorus recovery efficiency.
- Thermal pretreatment methods have been selected as optimal for most applications.
- Innovative research has been identified: removal of heavy metals, bioavailability.

## 1. Introduction

The area of phosphorus recovery from wastewater and bio-based waste is attracting considerable interest because of the depleting deposits of non-renewable resources, which are additionally related to the geopolitical problem. Of particular importance is the fact that phosphorus is present at relatively high levels in municipal wastewater and waste of biological origin, from which it can be recovered. This paper provides an overview of currently available methods for recovering phosphorus from secondary raw materials. This work includes a comprehensive and extensive review of the technologies available over the last 10 years. The aim of this work is to broaden our knowledge of phosphorus recovery, so that the technologies developed by scientists would soon be implemented in production practice. This study provides important information on a review of the available methods with an indication of their technological and economic limitations.

Examples of renewable sources of phosphorus are municipal wastewater from which phosphorus can be recovered by biological (Biological Excess Phosphorus Removal) or chemical (precipitation) methods. Therefore, sewage sludge, especially from the third stage of municipal wastewater treatment plants, can be used for the recovery of phosphorus compounds. Other secondary raw materials are bones-containing waste (slaughterhouse waste), i.e., the hydroxyapatite form of this element. Other bio-based phosphorus-containing wastes are crop residues, livestock production (manure, slurry), and waste from the food industry. Each time, the recovery of phosphorus requires the use of appropriate processing to obtain a fertilizing form of phosphorus that will be soluble in the soil solution.

The present work has been divided into the following parts. The part ‘Phosphorus management’ of this paper introduces sources and forms of phosphorus in the environment, describes environmental aspects, and characterizes the use of phosphorus over the years. The section ‘Phosphorus recovery technologies and trends from a 10-year perspective’ describes the available technologies for the recovery of phosphorus from wastewater, biowaste, and sewage sludge, characterizes biological and chemical treatment, the sorption process, and thermal treatment. This is followed by the section ‘Guidelines for a practical approach’, which addresses the barriers that limit the implementation of elaborated technologies and defines the approach to standardization of products obtained from secondary resources. The next section considers ‘Future perspectives’, which analyses future work that needs to be done to successfully implement these technologies in practice.

There are several reviews in the literature on various aspects of phosphorus recovery from various materials. However, most of these works are devoted to a fairly narrow subject matter, individual phosphorus-bearing secondary materials, or individual phosphorus recovery techniques. On the contrary, this work provides a broad overview of currently available technologies in a broad sense. The work discusses the barriers that prevent many of these technologies from being implemented in practice. To show the uniqueness of this literature review, the characteristics of reviews that have already appeared on a similar topic are presented. Carrillo et al. focused on the recovery of phosphorus from wastewater using hybrid technologies [1]. Few researchers have addressed the problem of developing countries. Chrispim et al. conducted a critical review of phosphorus recovery from municipal treatment plants [2]. Daneshgar et al. provide an overview of methods to overcome the phosphorus crisis. The review article describes ways to protect renewable raw materials and possible alternative technologies [3]. Guo et al. draws on an extensive range of sources to provide an overview of emerging technologies for purification and recovery [4]. In their paper, Kwapinski et al. reported technologies for the recovery of phosphorus from heat treated sewage sludge [5]. The study by Li et al. offers an analysis of the recovery of phosphorus from wastewater using membrane technologies [6]. Roy et al. draw attention to ecological engineering as a general approach to phosphorus recovery [7]. Tan and Lagerkvist (2011) described how phosphorus is recovered from ashes from biomass combustion in a work done 11 years ago [8]. Wilfert et al. reviewed the knowledge of phosphorus and iron in relation to methods for recovering phosphorus from wastewater [9]. Wu et al. published a review on the recovery of phosphorus from wastewater in the form of vivianite [10]. Yang et al. developed a review of biological phosphorus recovery [11]. In turn, Yu et al. characterized the possibilities of recovering phosphorus from sewage sludge, taking into account species, fractions, and characterization [12]. In their 1998 paper, Morse et al. reported on phosphorus removal and recovery technologies. It is the work that most broadly describes this issue and is closest to the scope of this work [13]. However, it was written 24 years ago. The present work is a broad overview that summarizes the last 10 years of research on phosphorus recovery from wastewater and bio-based waste technologies.

The first methodology in which phosphorus was isolated was related to a renewable resource, which was urine. At present, it can be said that we are going back to the beginnings of research on this element. Phosphorus (P) was discovered in Hamburg around 1669 by an alchemist, Dr. Brandt, who tried to convert urine (golden liquid) into gold [14]. Until 1750, phosphorus was a rare and expensive material. At this time, P has found applications mainly in medicine. In the nineteenth century, its production was carried out mainly from bone materials. The first industrial technology was developed by Albright and Wilson Plc [14,15]. On an industrial scale, since 1860, mineral raw materials have been used to produce this element. It soon became clear how important phosphorus was. On the one hand, the role in agronomy was recognized; on the other, it was used in the production of phosphor bombs, as ironically used for the first time in Hamburg by the Allied forces in World War II [16]. Phosphorus is an element that readily creates chemical compounds. It often occurs in the form of 17 phosphates [17], which in the soil are bound to particles and are not readily available to plants until the soil is saturated or there are no soil particles with which phosphorus can bind. The result is a high demand for phosphorus fertilizers [18].

Phosphorus is an essential element for the growth of all living organisms; therefore, it is necessary to produce food. Although it is only the eleventh most common element on Earth, phosphorus never appears in pure form, and it always remains bound in compounds, e.g. in the form of phosphate rock. Importantly, most of the phosphorus in the soil is not available to plants, so the soil requires the addition of nutrients. The sources of this element are most often non-renewable raw materials extracted at an increasing rate to meet the demand for mineral fertilizers. Globally, mineral fertilizers are responsible for the supply of 80% phosphorus, the remaining 20% are detergents, animal feed, and others [19]. More than 30 countries extract phosphates for commercial purposes. The first 12 countries provide 95% of the phosphorus on the market. Currently, Morocco remains the leading supplier of this element (about 50%). In addition, the country<apos;>s resources are estimated at 60% of all resources on Earth. According to various sources, global deposits will last 90 to 130 years. However, analysts agree that the continuous production of phosphorus compounds will soon lead to deterioration in quality and an increase in prices for this raw material [20]. As a result, there has been a growing amount of work on recovering this valuable element from biological waste in recent years. This work summarizes the methods commonly utilized in P recovery by indicating their potential for the future, optimal parameters, and development paths. In our opinion, gathering the latest technological achievements in one review may be a milestone and a breakthrough in the implementation of phosphorus recovery technologies.

## 2. Phosphorus in the environment

### 2.1. Sources and forms of phosphorus in the environment

Phosphorus compounds discharged into the aquatic environment occur in dissolved, precipitated, or adsorbed form due to physical, chemical, and biological processes [21]. Most of the forms of phosphorus found in the environment are inorganic phosphates and organic phosphorus that can be found in the form of phosphate ester in plants or soils and organic, renewable sources from biological waste materials [22,23].

Phosphorus has broad industrial uses. Examples of inorganic phosphates used in industry are tripolyphosphates – three-chain phosphorus compounds, calcium and ammonium orthophosphates and polyphosphates (fertilizers) [24]. Organic phosphates are used in insecticides, plasticizers, and surfactants. In the environment, both organic and inorganic phosphates are biologically and chemically degraded to form orthophosphates as the final degradation product [22,25].

**Figure 1.**
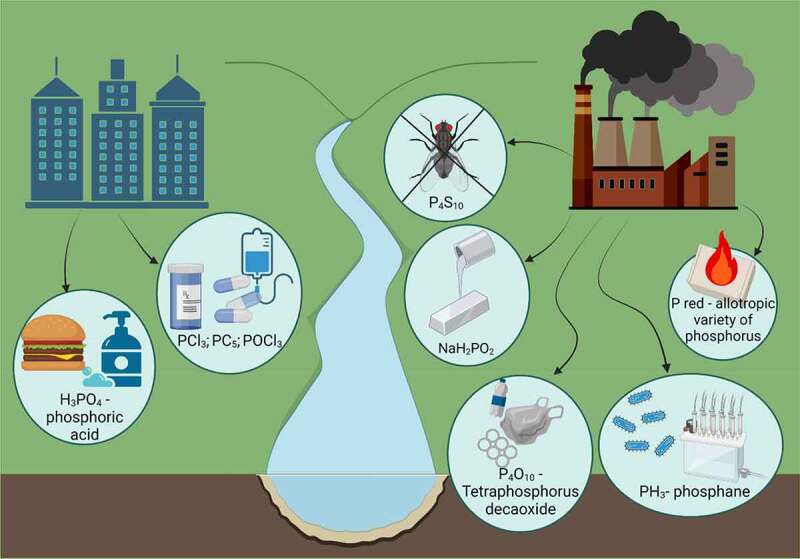
Sources of phosphorus and its application in daily life (from left): food and cosmetic industry, pharmaceutical industry, biocides industry, metallurgical industry, plastics industry, pyrotechnic industry [21,22,24,26].

Figure 1 indicates potential sources of phosphorus, which is widely applied in agriculture as feed additives or as a fertilizer component [26]. It explains its increased consumption by individuals, although various forms of phosphorus result in its increased occurrence in other aspects of everyday life. Phosphorus chlorides are used for the production of pharmaceuticals and electrolytes for individual use. However, other forms are also used in heavy industry as plastics additives, extractors, flotation agents for nickel plating, pyrotechnics, or ligands** [27]

**Figure 2.**
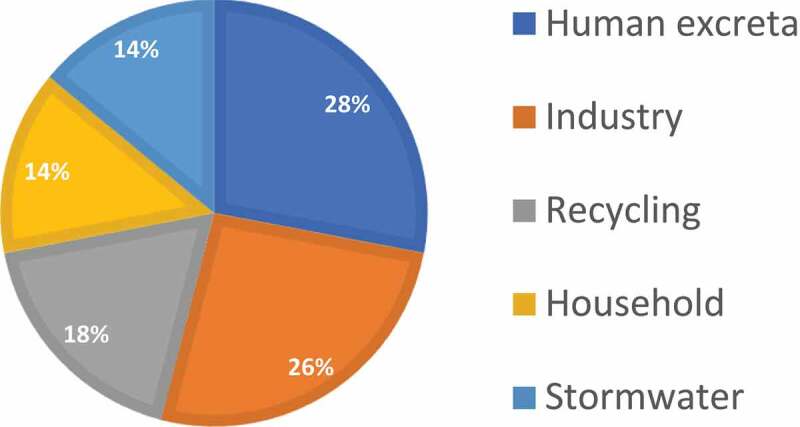
Sources of phosphorus in wastewater[100].

In wastewater, waste containing phosphorus is found in varying proportions Figure 2. The largest source of them is human excreta and industrial wastewater. Other groups, i.e. recycling, household, and stormwater are also present in significant proportions.

**Figure 3.**
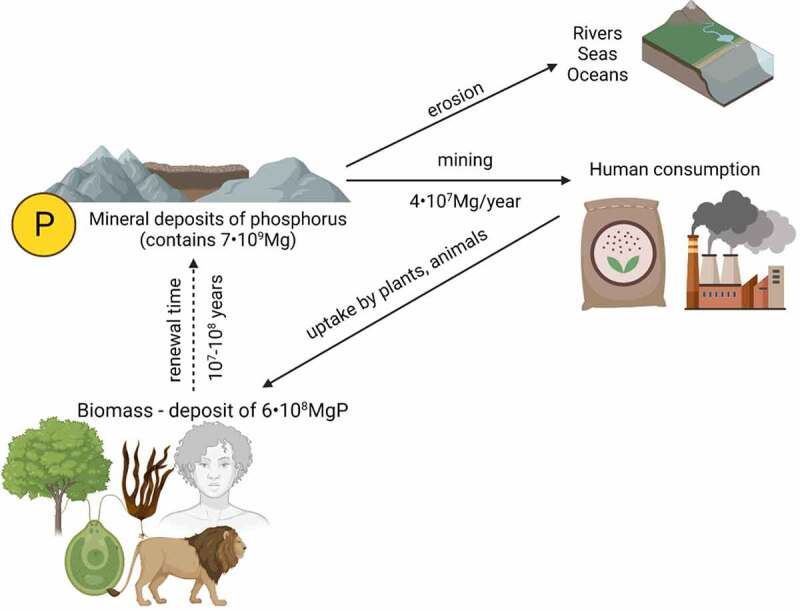
Life cycle of phosphorus mineral deposits [28].

Wastewater is only part of the phosphorus cycle. Their overall relationship with the environment is shown in Figure 3.

Due to the variety of forms of phosphorus, it is also important to note which form is present in each waste [29]:

-orthophosphate (agro-industrial wastes, swine manure, sludge from WWTPs)

-pyrophosphate (crop biomass)

-polyphosphates (sludge from WWTPs)

-phytic acid (agro-industrial wastes)

-phosphate diesters (crop biomass, agro-industrial wastes)

Wide usage presents the importance of phosphorus in everyday and industrial life and the necessity of its recovery from the environment.

### 2.2. Wastewater and bio-based waste as renewable phosphorus sources

The waste resources with the highest phosphorus content are wastewater, sewage sludge, animal production residues, and, to a lesser extent, waste from the agri-food sector (Table 1). In most cases, apart from phosphorus, other valuable streams can be recovered, including nitrogen, carbon, and energy.

**Table 1.** Phosphorus content in selected biowaste

Biomass and biowaste	P content	References
Liquid fraction swine manure	0.203 g/L	[148]
Dairy manure	4.10 g/kg – 18.3 g/kg DM	[131]
Dairy manure slurry	67.5–101.0 mg/L	[131]
Pig manure	1.9 g/kg	[152]
Poultry litter	13.6 g/kg	[30]
Slaughter waste	1.79 g/kg DM	[31]
Cattle bones	104 g/kg DM	[55]
Pig bones	93.6 g/kg DM	[55]
Poultry bones	85.2 g/kg DM	[55]
Fish bone ash	172 g/kg	[32]
Chicken bone ash	155 g/kg	[32]
Beef bone ash	142 g/kg	[32]
Sewage sludge ash	80 g/kg	[44]
Sewage sludge ash	88.4 g/kg	[33]
Sewage sludge	25.68 g/kg	[34]
Sugarcane waste	5.30 g/kg	[30]
Cabbage waste	0.26 g/kg	[30]
Food waste	4.2 g/kg	[35]

#### 2.2.1. Wastewater

Wastewaters are the source of different macro and microelements, organic matter and heavy metals. The nutrient value of wastewaters is characterized by a low phosphorus concentration but a high volume, since one EU citizen produces around 200 L of wastewater per day with an average concentration of 10 mgP/L, the potential source of P in wastewaters generated by the capita is estimated at 1.8–2.5 g P/day or ∼1 kg P annually [36–38]. It was determined that in 2013, an average of 330 km^3^/year of wastewater worldwide was generated [39].

#### 2.2.2. Sewage sludge

Sewage sludge (SS) is a large waste, estimated to represent up to 2% of the treated wastewater volume [40]. The daily amount of sludge generated as dry matter is 60–90 g per EU inhabitant [41]. Depending on the region, the production of sewage sludge may differ in Europe and in the USA, production amounts to 8909 (2010); 6514 (2004) thousand tons, respectively. Although in China, Japan and Brazil, it is estimated to be 2966 (2006); 2000 (2006); 372 (2005) thousand tons, respectively. This indicates large differences in the production of sewage sludge mainly due to poorly developed wastewater treatment plants in less developed countries [39]. Sewage sludge is one of the wastes containing the highest levels of phosphorus. Phosphorus is stored in cells of microorganisms as polyphosphate that is used in the removal of biological excess phosphorus or in the form of insoluble phosphates [42]. Approximately 90% of the total phosphorus load in wastewater is collected as activated sludge [36]. The phosphorus content in sewage sludge and its ashes reaches 1–3 [43] and 5–10 wt% (11–23 wt% P_2_O_5_) [44], respectively. Tertiary treatment sludge contains high levels of phosphorus but lower levels of heavy metals; however, it includes a smaller amount of organic compounds, which are essential in agricultural applications [45]. Direct use of SS for agricultural purposes is impossible due to pathogenic microorganisms and various micropollutants (pharmaceuticals, hormones, organic substances, and heavy metals), which pose a threat to the environment and human health. Sewage sludge requires pretreatment based on its decomposition or thermal treatment [46,47]. The sewage sludge can be digested or incinerated and the resulting products (digested sewage sludge, DSS and sewage sludge ash, SSA) can be transferred for further processing. DSS and SSA contain less organic compounds compared to raw sludge. Ashes from SS incineration include phosphorus and toxic metals such as lead, nickel, and cadmium in higher concentrations than incineration with SS. Their presence makes it difficult to use this material, and co-incineration with other waste, including municipal waste, should be avoided [48].

#### 2.2.3. Animal manure

The European Union produces more than 2 billion Mg of manure per year, which contains more than 5 million Mg of P_2_O_5_ [49]. This is only one-tenth of the world<apos;>s production, where the estimation assumes total production from 15–20 billion Mg of manure per year [50]. Animal manure is undigested food waste containing large amounts of organic matter, nitrogen compounds (uric acid from urine, organic nitrogen from feces), and phosphorus (mainly phytic acid as a residue of a cereal-based diet). Manure also contains a number of undesirable components, such as hormones, antibiotics, and pathogens, which inhibit its direct use as a fertilizer. Poultry litter is a material that contains manure and feed residues, feathers, litter, and water residues. However, it contains important fertilizer nutrients, which are in incorrect proportions (N/P) as for fertilizer use [51]. Direct application of manure to arable fields can contribute to increased leakage of components into surface water, resulting in its pollution, the release of volatile nitrogen compo or insufficiencies soil aeration [52]. The manure slurry is also not suitable for long-distance transport due to its high cost. Animal manure varies in composition, depending on the species and age of the animal, the type of diet, the amount of water supplied and even the climatic conditions [53,54].

#### 2.2.4. Slaughterhouse waste

Annually, more than 51 million Mg of livestock is slaughtered in EU-28 [55]. The slaughterhouse waste from the meat industry is mainly heads, limbs, bones, blood, offal, and fat, representing almost one-third of the animal<apos;>s weight [56]. Animal bones, rich in hydroxyapatite, have the highest phosphorus content (up to approximately 10% of dry matter) [55]. The slaughter residues are processed into a powdered form, a meat and bone meal. As a consequence of the ban on using meat and bone meal directly as animal feed and organic fertilizers, it is mainly incinerated and its ashes are an excellent source of phosphorus for fertilizer use [57].

### 2.3. Environmental aspects

Phosphorus has a contradictory nature. It is essential to sustain life, but at the same time destructive in excess in the aquatic environment, being one of the paradoxes of nature. Eutrophication involves the enrichment of freshwater and marine systems with nutrients, particularly nitrogen and phosphorus. In freshwater reservoirs, phosphorus is often a limiting nutrient, which reduces growth in various ecosystems [58,59]. That is why, in the case of the discharge of excessive amounts of phosphorus through agricultural leachate or municipal sewage, it leads to serious problems of water quality deterioration. Emerging algae bloom changes aquatic ecosystems and causes the death of many fish and other aquatic organisms. Dying algae biomass causes a decrease in oxygen concentration in deeper water layers and sediments. Eutrophication has been recognized as a serious global problem [60].

**Figure 4.**
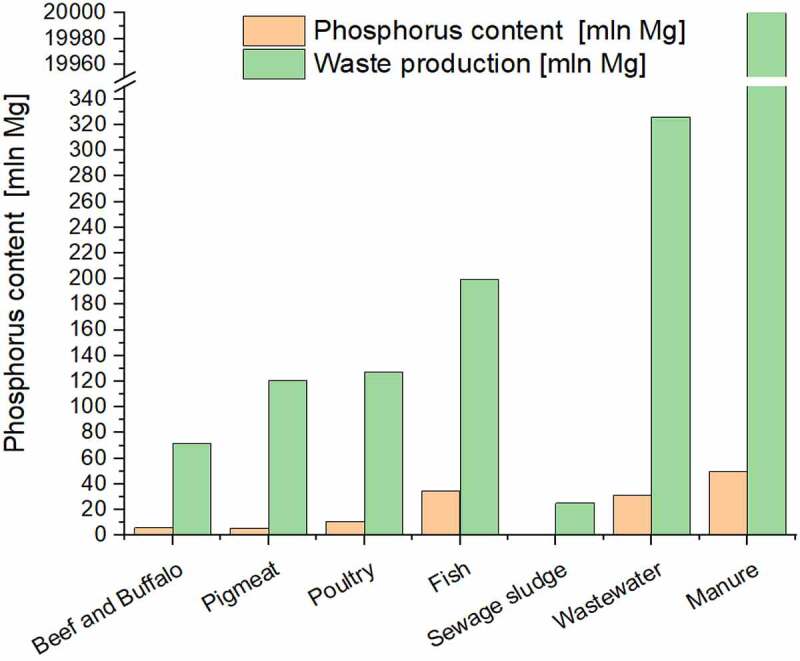
Potential for the recovery of phosphorus from animal waste. Waste phosphorus sources and the amount (in Mg) of waste phosphorus they contain [39,63–70].

Each individual discharges 2 g of phosphorus into the wastewater every day. Phosphorus discharged into wastewater is a danger to water reservoirs (eutrophication). An important option is the recovery of phosphorus from wastewater. Sewage is a renewable source of this element. It is estimated that 250,000 Mg of phosphorus in wastewater is produced annually in Western European countries. This is comparable to the industrial demand for phosphorus. Manure is an additional significant source of P. Therefore, the recovery of phosphorus from municipal wastewater and agriculture is an opportunity to significantly reduce phosphorus consumption from nonrenewable sources. This idea aligns with the sustainable development policy [61]. However, to implement the valorization of phosphorus on the industrial scale, the policymakers should introduce laws and subsidies to support fertilizers derived from renewable resource bases [62]. The potential of biological waste in terms of phosphorus content is shown in Figure 4.

## 3. Phosphorus recovery technologies and trends from 10 years perspective

### 3.1. Wastewater streams

The recovery of phosphorus compounds from wastewater treatment plant can be realized from liquids, slurries, or mixture of them like secondary treated effluent after biological treatment, anaerobic digester supernatant, sewage sludge and its derivatives such as ashes [37,71–73]. The challenges for ideal phosphorus recovery are different flows of wastewater with changeable concentrations of P compounds, dissolved or biologically and chemically bounded forms of phosphorus, high recovery rate and concentration of pollutants, as well as good quality of the recovered product [74–76].

Phosphorus recovery technologies from the wastewater streams are mainly used on site and are based on crystallization and precipitation. By the addition of compounds of Mg or Ca, the dissolved phosphates will be recovered in the form of the magnesium ammonium phosphate MgNH_4_PO_4_6H_2_O (MAP, struvite) or calcium phosphates (CaP): CaHPO_4_2H_2_O (brushite), Ca(H_2_PO_4_)_2_, hydroxyapatite (HAP) or octacalcium phosphate Ca_8_H_2_(PO_4_)·6.5H_2_O (OCP). The main difference between these methods is the response time. The precipitation is much faster, but the product obtained in this process has an amorphous structure. Crystallization occurs more slowly, but the resulting product has a crystal structure, making it more soluble and more valuable fertilizing material [37,77–79]. Recovery of P as vivianite (Fe_3_(PO_4_)_2_8H_2_O) is also considered due to its natural occurrence and predictable economic value [10].

Struvite crystallization is one of the well-known and developed processes, with wildly recognized and discussed parameters of production from the different feedstocks, in different reactors and process combinations reviewed in many publications [74,80–85]. The conclusions drawn by Li et al. based on 1424 papers reveal some crucial factors, listed in Table 2, influencing struvite technology: pH, temperature and mixing, magnesium addition and seeding, disturbance variables like foreign ions or organic matter [74].

**Table 2.** *Factors influencing struvite crystallization* [131]

	Optimal range	Positive influence with parameter rising	Negative influence
pH	8–10.5	Higher P removal efficiency (over 9), growth rate (9.5)	Purity (over 9), crystal size (10.5), solubility, Zeta-potential
Temperature and mixing	15–35°C, 160 rpm	Growth rate (50°C), altered crystal structure, saturation index, solubility, P removal, nucleation rate	Purity, sizes, pK sp, turbidity, induction time
Magnesium addition	Mg:P molar ratio 1–2	P removal, size, supersaturation	Increase in dosage costs
Seeding	Low- high concentration of different seeds	Crystal growth, size, P removal, and the crystallization rate	Sedimentation, induction time
Foreign ions	Ca:Mg 0.5	P removal, induction time, P distribution, size, the crystallization rate	Size, purity, fertility induction time, P removal

An important crystallization parameter is the appropriate Mg:N:P molar ratio for struvite of 1:1:1 and pH value 7.5–9. The proper pH of the reaction system is achieved by adding NaOH or CO_2_ stripping (aeration of the system to strip CO_2_) [37,74,86]. In the case of the precipitation, the process takes 1 h, pH range is between 8.5–9 and molar ratio Mg:N:P for struvite is 1.5:1:1 [81]. The crystallization process is carried out mainly in fluidized bed reactors or stirred reactors. Research on phosphorus recovery from wastewater was also carried out in addition to crystallization and precipitation. The possibility of using the ion exchange process to remove and recover phosphorus, where phosphate ions are retained on the anion exchanger and ammonium ions on the cationite, was also investigated. High purity struvite is precipitated from solutions after regeneration of ion exchangers [87]. Anaerobic membrane bioreactors [88], ion exchange and adsorption, magnetic microsorbents, reactive filtration, electrodialysis, biochemical and electrochemical systems [89,90], forward osmosis [91] or Integrated Constructed Wetlands (ICW) [92] were also investigated [88,89,91,93–95].

**Figure 5.**
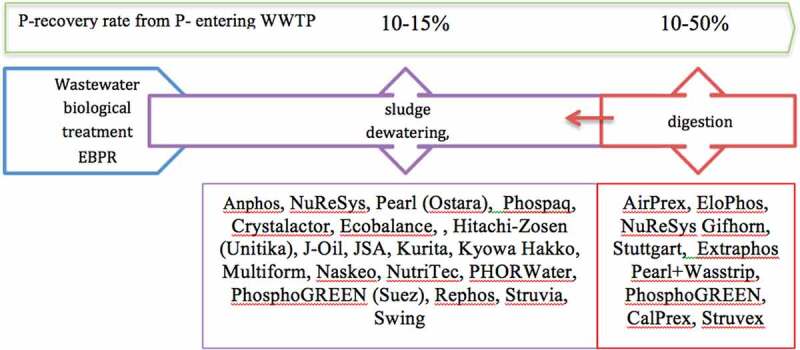
Possible options for P recovery from wastewaters.

In principle, there are four possible options for the recovery of phosphorus from wastewater treatment facilities that differ significantly with respect to recovery efficiencies. Direct recovery of struvite after biological stage with enhanced biological phosphorus removal (EBPR) has very low efficiencies between 10–15% of P from raw wastewater input, while P recovery after sludge treatment (dewatering and digestion) reaches 10–50% (average up to 20%) of wastewater P input Figure 5, 50% recovery can be achieved by force dissolution or other processes such as sludge hydrolysis. Only the recovery of phosphorus-rich ashes is characterized by a high recovery potential of 85–95% [71,96].

**Figure 6.**
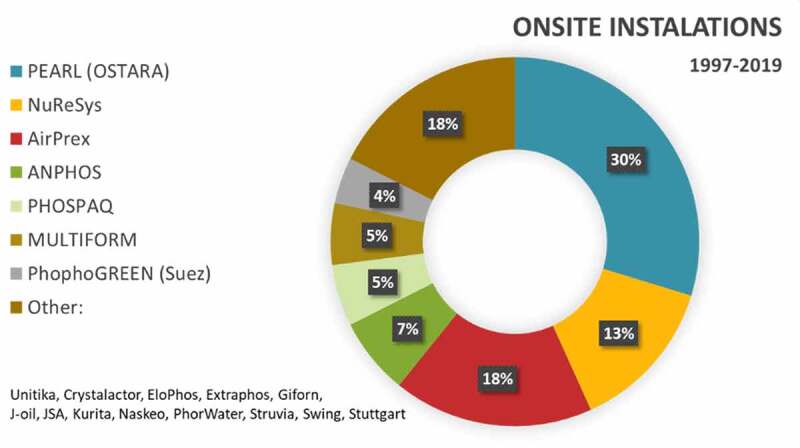
Phosphorus recovery installations from wastewater streams with commercial phosphorus recovery facilities [96–98].

The oldest process was developed in 1970 to prevent the growth of struvite in installations in the sewage treatment plant. Today, the most common methods of phosphorus recovery from the wastewater streams are based on the crystallization process. The oldest installations like KURITA, Hitachi-Zosen (Unitika) and JSA were built in Japan in 1997 and 1998, while REPHOS, Kyowa Hakko and PHOSPAQ operate from 2006. From a 10-year perspective of 74 installations operated worldwide in 2019, only 13 installations were built before 2010, while 61 installations (82.4%) were built between 2010–2019 (Figure 6). Pearl technology is one of the most often used today with 22 installations in the world. Ten NuReSys installations are applied for dairy industry, French fries production and municipal wastewater treatment plants in Belgium, Netherlands and Germany. 13 AirPrex installations work in Germany, the Netherlands and China [87,97,98].

The main parameters of phosphorus recovery technologies were characterized in Table 3

**Table 3.** Characteristic of selected phosphorus recovery technologies from wastewater streams

Technology	Type of P-recovery	P-recovery rate from P- entering WWTP [%]	P-removal efficiencies from influent [%]	Reactor type	Product	Operating plants	Installations capacity [kg of product/day]	References
PHOSPAQ	Type I	10–15	80	Mixed stirred tanks	struvite	4	80–1200	[2–37–71–83–85––107]
ANPHOS	Type I	10–15% (municipal) 90% (potato industry)	63–90	Mixed stirred tanks, batch process	struvite	5	400–1800	[2,71,83,85,96,105-107]
NuReSys	Type I	10–15	85	Mixed stirred tanks, continuous process	Struvite BioSTRU	10	55–2000	[37,71,83,85,96,105-107]
Ostara PEARL™	Type I	10–15	85	Fluidized bed reactor	struvite	22	325–6350	[37,71,81,83–86,88,89,91–94,186]
PhosphoGREEN (Suez),	Type I	10–15	80–90	Fluidized bed reactor	Struvite PhosphoGREEN	3	230–270	[2,71,96,107]
AirPrex	Type II	10–50	80–90	Outflow stream form anaerobic digester, Cylindrical reactor, Wasstrip, CalPrex	Struvite Berliner Pflanze	13	500–4550	[2–37–71–82–96–99–]
Gifhorn	Type III	50	90	Wet chemical process, mixed stirred tanks, ammonia striping	struvite 41% solution ammonium sulphate	1	580 1300	[87,97,98]
Stuttgart	Type III	50	80–90	Wet chemical process, chamber filter press batch or semi-batch	struvite	2	63–252	[37,83,84,186]

Type I- from the sludge liquor after the sludge dewatering unit form EBPR processes

Type II- P precipitation upstream from the sludge after dewatering

Type III – P precipitation downstream from the sludge

Fluidized bed reactors are used in Ostara PEARL™ technology developed at the University of British Columbia in Vancouver (Canada), PhosphoGREEN proposed by SUEZ and Multiform. An innovative element of Ostara technology is the patented cylindrical reactor with decreasing diameter, as well as small product particles recycled into the process for better crystallization [2,3,37,71,85,96–98,100–106]. Multiform technology uses a conical-shaped fluidized bed reactor to crystallize lower grade struvite with 2.5 times lower costs than Pearl [2,83,96,100]. Ostara working reactors can recover 325–6350 kg of struvite per day, while PhosphoGREEN installations only 230–270 kg [2,71,96,107].

PHOSPAQ, ANPHOS® and NuReSys uses mixed stirred tank reactors with the capacities between 80–2000 kg of struvite per day. pH adjustment is accomplished by stripping carbon dioxide in the case of the first two processes, while NaOH is added in the third. Struvite crystallization by addition of MgO or Mg(OH)_2_ takes place in the second reactor. PHOSPAQ uses a hydrocyclone and a screw press for final product treatment. Four PHOSPAQ installations and five ANPHOS® were built in 2006–2016 to treat wastewater from the potato industry and sludge dewatering liquor in the Netherlands, UK and Italy [2,71,83,85,96,105-107]. Ten NuReSys installations work in Belgium, Netherlands and Germany, and BioSTRU is certified as fertilizer in Belgium [2,37,71,83,85,96,100,105–107].

AirPrex®is a technology developed by Berliner Wasserbetriebe (BWB) in cooperation with Technische Universität Berlin [108] that uses nutrients rich sludge from the digestion chamber. As in the PHOSPAQ, an increase in pH is achieved by removing carbon dioxide by aeration. The unique design of the reactor allows the struvite particles to precipitate to the cone-shaped bottom, from where this product can be discharged continuously. The degree of phosphorus recovery for this technology in relation into the quantity introduced to the sewage treatment plant is approximately 7–22%. This technology is also included in the group of phosphorus recovery methods from sewage sludge when combined with the Lysogest® technology, which relates to the recovery of phosphorus from sewage sludge subjected to thermal hydrolysis. CalPrex and AirPrex capture 50% phosphorus entering treatment plants as brushite (CaHPO_4_ · 2H_2_O) and struvite (MgNH_4_PO_4_ · 6H_2_O) [2,37,71,82,96,100,102,105–107].

Ostara<apos;>s PEARL™ technology is one of the most developed with 22 installations, the largest capacities, and registered product, while AirPrex technology with the highest P-recovery is one of the most complex and growing.

First-generation installations for P precipitation show a typical recovery of about 10–20% and consist of the additional reactor after the digester or after the decanter. Around 60% of the new municipal wastewaters will be supplied with such systems to recover 11,880 Mg P annually. Second-generation installations are equipped with additional treatment units in the sludge line prior to the anaerobic digester, such as WASSTRIP or CAMBI- thermal hydrolysis, to lift P-recovery efficiencies up to 50%, with economic benefits by reduction of sludge volumes and better dewatering. They can be installed only in large WWTP (>150,000 population equivalent) due to the higher investment costs and according to estimates close to 19,800 Mg of P could be recovered [71,97,104,109].

The research and application of phosphate salt crystallization technology throughout the world is the best proof that it is a good solution for phosphorus recovery. Some gaps were identified, such as increasing P-removal efficiencies, lowering production costs, a dosage of different magnesium sources for cost reduction, improvements in struvite quality or clear recommendations for seed selection and dose, as well as dynamic models that describe struvite crystallization instead of those based on thermodynamics [75,96].

#### 3.1.1. Struvite safety and recovery potential

Regulation (EU) 2019/1009 of the European Parliament and of the Council of 5 June 2019 providing rules on the making fertilizing products available on the market of the EU introduces eleven new component material categories (CMC 1–11), which can be used for fertilizer production, including waste materials. Additional categories such as precipitated phosphate salts & derivates (CMC 13) proposed by the STRUBIAS group were not included, but after 15 July 2019, the Commission shall evaluate struvite and if the criteria are met, the delegated acts will be adopted to include these materials as CMCs and open EU market [71,110].

The parameters that precipitated phosphate salts that must be met as a component material for the fertilizing product are as follows: minimum 16% P_2_O_5_ content in dry matter, organic C content lower than 3%, maximum 10% of the sum of elemental Al and elemental Fe, no presence of *Salmonella* spp. in a 25 g sample and no presence of *Escherichia coli* or *Enterococcaceae* in a concentration of more than 1000 CFU/g of fresh mass, no presence of Clostridium perfringens in a concentration of more than 100 CFU/g of fresh mass, no presence of viable *Ascaris* sp. eggs in a 25 g of fresh mass. The final product should be free from visually detectable physical impurities such as organic materials, stones, glass, and metals greater than 2 mm to <0.3% and should be stored in dry conditions [71].

**Figure 7.**
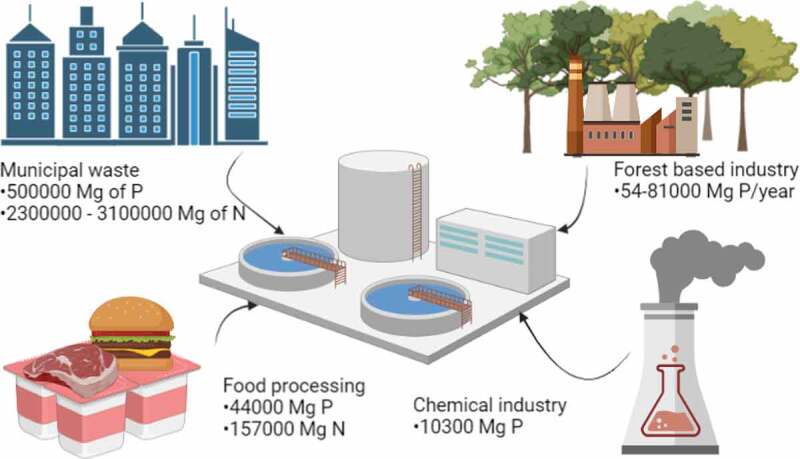
Potential for the recovery of phosphorus from wastewater, industries along with the phosphorus and nitrogen content [110].

According to estimates, in 2030, around 60% of recovered phosphate salts will be derived from municipal wastewater and 39% from manure. Other wastewater streams useful for P-recovery are industrial waste streams from the potato or dairy, liquid stable solutions from manure and other livestock treatment rich in P and ammonia [15,17,87]. The potential for P recovery from municipal and industrial wastewater is estimated at 635,300 Mg of P annually (Figure 7); today only 15,000 Mg of struvite per year is traded in the EU with individual permits (CrystalGreen, BioSTRU and PhosphoGREEN). Despite the high potential for phosphorus in wastewaters, all products precipitated from their streams could reach only 99,000 Mg of P per year due to low recovery rates and lack of recovery installations [71,110].

The recovery costs of phosphate salts from wastewater are estimated as 2–8 euro per kg of phosphorus [36]. On the other hand, the annual costs for implementing phosphorus recovery processes were estimated at 2 to 6 euros per capita [36]. Struvite obtained from wastewater can be sold to fertilizer companies or distributed on the market at a price of 188–763 euro/Mg [2,111,112].

The research focused on LCA analysis underlines the need for a further study of the environmental impact of the recovery of phosphate salts from wastewaters streams, especially outside Europe, as the well as assessment of full-scale installations in terms of further cost reduction [102,113–115].

If the global mass flow of phosphorus is considered, then wastewater includes only 5% of anthropogenic flow, its recovery appears to be unimportant. However, when 80% of our population lives in cities, such technologies are needed and should be promoted. The potential for P recovery from municipal and industrial wastewater in the EU is estimated at 635,300 Mg of P annually, but it is still used on a small scale.

Phosphorus recovery technologies from wastewater are well researched and proven, as the oldest struvite precipitation process was developed in 1970 and is commonly used in the last 10 years, when 82% of installations were built.

WWTP phosphorus (municipal and industrial) can be recovered in technologies with the efficiency of 10–90%, directly after the biological stage with enhanced biological phosphorus removal (EBPR), after sludge treatment (dewatering and digestion) with implemented force dissolution or hydrolysis of the sludge. It is very important to recover phosphorus after sewage sludge treatment, otherwise it may end up back in EBPR, making the effort of this method pointless. As a final recovery product, ammonium magnesium sulfate – struvite is produced, recognized as an effective fertilizer and called ‘next generation fertilizer’ due to its high water solubility. Advantageously, struvite crystallization is also used as a purification step and is widely promoted to improve the technical efficiency of wastewater treatment technologies. The main advantage of biological methods is the cost of their operation. For large volumes and low phosphorus concentrations, the purchase of precipitation reagents may not be economically viable. Due to the use of biological methods in specific cases, where the necessity of a dozen or so days of liquid storage is not a problem (e.g., storage tanks for waste, artificial retention basins), it can be a beneficial method. The biosorption method is also applicable as a preconcentration of phosphorus from very dilute sources for further recovery. Under specific stressful conditions, microorganisms can accumulate up to 8% phosphorus in dry biomass. It can then be recovered both by thermal methods and by precipitation using anaerobic digestion [116].

Ostara<apos;>s PEARL™ technology is one of the most developed with 22 installations, the largest capacities, and registered product, while AirPrex technology with the highest P-recovery is one of the most complex and growing. Estimated P -recovery cost from wastewater streams ranged from 2–8 euro/kg of phosphorus [36]. Struvite is a recognized marketable product known as CrystalGreen, BioSTRU, Berliner Pflanze and PhosphoGREEN, sell at a price from 188–763 euro/Mg.

### 3.2. Bio-based waste

#### 3.2.1. Biological treatment

*Composting*

The simplest process of bio-based waste treatment is composting. Composting promotes the mineralization of organic phosphorus to inorganic forms with better bioavailability. This process also reduces the volume and quantity of water in the material, but is associated with the loss of nitrogen compounds (volatile ammonia) [117]. The high water content is often problematic in bio-based wastes (manure, sewage sludge), requiring the utilization of various fillers to reduce moisture. Natural organic materials such as perlite or bentonite are often used for this purpose[118]. The composting of pig manure supported by treatment with housefly larvae was found to achieve a similar effect without the use of additives. The resulting product contained over 30% more nutrients compared to composting with fillers [119].

During aerobic transformation of sewage sludge, the composting process can be significantly improved along with the reduction of odor emission by using co-composting with agricultural residues, mainly lignocellulosic materials. These additives contain less nitrogen and improve the C/N ratio in the mixture which significantly increases the efficiency of microbial transformation and also affects the reduction of unpleasant odor emission [120]. Thus prepared stable compost free from pathogens can be used as fertilizer [121].

##### 3.2.1.1. Anaerobic digestion

Anaerobic digestion (AD) is a waste processing method with simultaneous production of energy (biogas) [122]. It is a popular method for volume reduction and sanitization of sewage sludge and animal manure. Phosphorus transformations in microbial processes are complex and strongly dependent on process conditions such as pH, presence of ions, particles, and physicochemical characteristics of the feedstock [123]. The AD process converts organic forms of nitrogen and phosphorus into inorganic derivatives such as NH_3_ and orthophosphates. Phosphates are found in digestate and are easily available for plants [124] and can be recovered from the liquid in the form of struvite [125]. It has been shown that the addition of calcium can have a beneficial effect on the isolation of phosphorus from swine manure, increasing its recovery from 60 to 74%, while improving its ability to be separated into high P content calcium phosphate granules (nearly 80%) [126].

In the AD process, as in aerobic microbial treatment, the C/N ratio is very important (preferably 20–30), so co-fermentation of manure with other biomass wastes is practiced, resulting in increased fermentation efficiency [127]. Additives are also used to accelerate fermentation, and pretreatment of the feedstock (chemical, mechanical, biological) is known to increase biogas production and nutrient recovery in the digestate [128]. The need for pretreatment arises in the case of sewage sludge, which on the one hand has the greatest potential as a feedstock for AD, but on the other hand is difficult to treat due to the high content of extracellular matter that needs to be pretreated in order to be available for microorganisms [40]. The solid fraction from the AD process can be an excellent source of phosphorus and nitrogen; by acid dissolution of the sludge after co-digestion of poultry manure and maize silage, 90% of these nutrients can be recovered, allowing complete utilization of all process streams in AD [129].

The main disadvantage of biological methods is the long process time, which can take up to a month. Due to the necessity of providing apparatus and space to run the process for such a long time, this is not a universal solution. Therefore, these methods are recommended in particular as a pretreatment because of the lack of reagents, the favorable bioavailability of phosphorus, and the possibility to obtain biogas.

#### 3.2.2. Chemical treatment

##### 3.2.2.1. Acid and alkali treatment

Raw manure, ash or biochar from the heat treatment of the manure can be extracted by utilization of acid treatment. Phosphorus is then successively recovered by precipitating the extract [117]. Rapid washing of solid waste with acid allows extraction of as much phosphorus as possible from the solution. This limits the possible losses of P from the waste material. The obtained solution is then subjected to the alkaline environment, where Ca-P precipitate is formed. The process is susceptible to the utilization of additives such as polymers, e.g. polyacrylamide. This allows for improving efficiency at a low cost [130]. Electrocoagulation can also be an effective separation of phosphorus microparticles from the solution [131]. A variety of phosphorus extraction methods from the solution indicate a possibility for adapting them based on the forms of P or the P content in the eluent. After sulfuric acid treatment, slaughterhouse waste and poultry feathers can be included in the composition of multicomponent fertilizers with high bioavailability for plants [132]. Acidic hydrolysis of manure helps to transfer phosphorus from the solid to the liquid phase, while reducing ammonia emissions to the atmosphere. The components of the liquid fraction can be selectively separated by bipolar electrodialysis [133]. This allows for the removal of potential heavy metal contamination of the fertilizer material. Recovery of phosphate from poultry litter can be carried out in two stages: acid and carbon dioxide treatment and precipitation in an alkaline environment with simultaneous aeration. With this method, more than 70% of phosphorus can be recovered in a short time (less than an hour)*** [134]. The article by Staro et al. (2016) evaluated potential waste from the meat industry, such as feathers, meat and bones, and poultry litter, to obtain fertilizer additives rich in macronutrients.

The application of heat treatment from 600°C to 900°C allows for the recovery of 30 to 170 g/kg of phosphorus. The highest values were obtained for 900°C and 3 hours of conducting the process. It was determined that it is possible to apply the resultant ash as a soil conditioning agent. Bergfeldt et al. (2018) indicated that the utilization of pyrolysis up to 500°C is the optimal method to obtain pyrochars that contain permissible heavy metal content, while maintaining a rich composition of nutrients [135]. The high solubilization of phosphorus in solutions simulating soil environment was determined by extraction in ammonium citrate 84.1% (450°C) and 90.7% in citric acid (500°C) for obtained pyrochars. The remaining compounds, such as Ca and K, showed similar bioavailability trends, which were evaluated in the pot tests. However, the authors have indicated that more systematic trials are required because the possibility of elevated levels of dissolved P can limit plant growth and its application.

**Figure 8.**
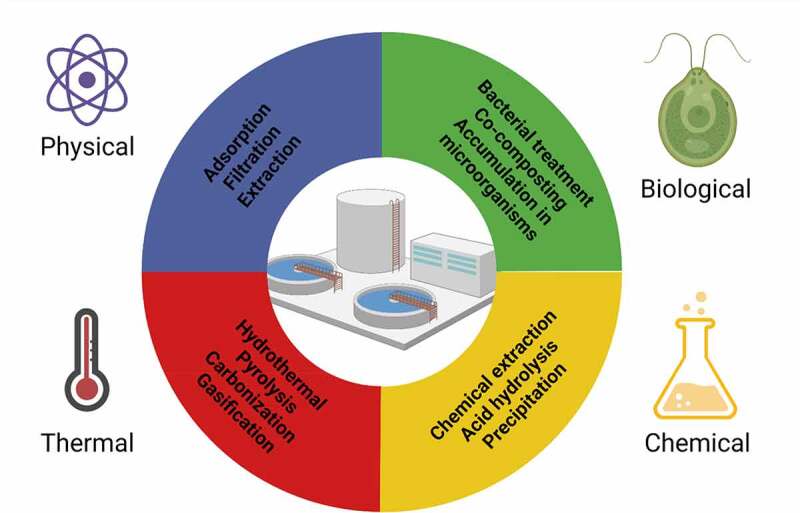
Summary of phosphorus recovery methods and their classification into four categories: physical – blue, thermal – red, chemical – yellow.

The phosphorus present in wastewater sludge is characterized by a low bioavailability to plants, so it is necessary to utilize processes enabling the separation of phosphorus from the sludge, including the most popular wet-process phosphoric acid and thermal methods. The breakdown of phosphorus recovery methods is presented in Figure 8. Both SS and SSA can be treated with a wet chemical extraction method (utilizing acid or alkali) in which the bound phosphorus is dissolved. A supernatant with a high P content is then further processed to separate phosphates from the solution. This method is more effective for DSS because it has a significantly lower organic content, approximately three times more phosphorus has been extracted compared to raw SS [136]. Simultaneously with the dissolution of phosphorus, heavy metal ions present in sewage sludge are transferred to the solution. Additional operations are necessary to separate heavy metal ions, i.e., their selective precipitation (usually in the form of sulfides), extraction, and ion exchange. After separating unwanted components, phosphorus can be isolated from the liquid phase by various methods, including precipitation, crystallization, ion exchange, and membrane techniques [36]. Phosphorus compounds are isolated mainly in the form of phosphoric acid, hydroxyapatite, struvite and vivianite (Fe_2_(PO_4_)_3_8H_2_O). Struvite can be recovered up to a concentration of 10 mgP/L in the solution, ensuring pH control and the proper content of magnesium compounds [48]. The quantity of P allows for utilization in the fertilizer industry. Vivianite is produced during anaerobic digestion of sewage sludge in the presence of iron [137]. The purity of the final phosphorus product, which can be used directly for agricultural purposes, is essential [138]. The removal of unwanted contaminants is relatively expensive as it requires additional reagents and processes. These costs should be kept as low as possible to recover phosphorus cost-effectively.

Anaerobic digestion has been evaluated in terms of phosphorus recovery from waste-activated sludge. The authors specified that different methods of material preparation, thermal process, changes in pH, zonation, and sonication were compared. As a result of the investigation, changes in pH at the level of 2 and 12 were indicated to obtain phosphorus solubilization at the level of 30% and 34%, respectively [139]. A study has shown that this is most likely the result of orthophosphate dissolution as a result of a change in pH. Anaerobic digestion has been found to release phosphorus from waste. However, the introduced pretreatment methods increased the level of mineralization of phosphorus during anaerobic digestion. The authors indicated that it is possible to separate the obtained phosphorus by introducing an additional in situ crystallization process. The pretreatment methods and their impact on the efficiency of phosphorus recovery were described. The article of Hu et al., (2021), presented the redirection of excess sludge to the following processes: anaerobic digestion, EDTA-anaerobic digestion, and ultrasound with EDTA-anaerobic digestion [140]. It was indicated that the highest degree of recovery (53.50%) was obtained for ultrasound method, which is noteworthy compared to other procedures where the result was less than 35%. It is also a much greater value than in the methods used in the study by Liu et al. (2019), that suggests the possibility of further modification or combination of these methods. However, all techniques present a high degree of recovery of phosphorus from the supernatant up to 94.49% for anaerobic digestion, resulting in obtaining struvite with a high purity of 85.14%. These papers allow for the conclusion that the methods of pretreatment of the material are of crucial importance in the production of fertilizing compounds with a small amount of metallic waste.

In wet-process technology, the addition of strong acid allows the solubilization of most phosphorus compounds, with the simultaneous release of metal ions. The most commonly used are strong inorganic acids, including sulfuric acid [141], nitric acid [142], hydrochloric acid [143], but also organic acids such as citric acid, lactic acid [144] and oxalic acid [145]. The strength of the acid utilized has a direct influence on the degree of phosphorus solubilization, at pH >2, it solubilizes less than 80% [37], while at the same time, less heavy metal ions are released into the solution. Metal ions remaining in the solution can be selectively separated from phosphorus in the Seaborn or Stuttgart approach [37]. Two-stage extraction involving the use of ethylenediaminetetraacetatic acid (EDTA) in the first stage and sulfuric acid in the second stage, resulting in much reduced release of metal ions in the acid environment [146]. Alkaline solubilization (NaOH) allows the solubilizer to lower the quantities of heavy metal ions from the material, except for zinc and arsenic compounds. All the others are not transferred to the solution [44]. Sequential extraction with acids and alkali causes more phosphorus is solubilized from biomass, the sequence in which reagents are used is also essential [147].

In the case of chemical methods, reagents must be used in proportion to the amount of phosphorus recovered, which, given the current trend towards a closed-loop economy, is an unfavorable solution. It is also necessary to separate toxic metals (depending on their presence in waste). The undeniable advantage of these methods is time (less than an hour) and efficiency (more than 90%), which in many applications may be the decisive advantage.

#### 3.2.3. Physical treatment

##### 3.2.3.1. Membrane technologies

Manure contains high levels of organic matter that is difficult to decompose, resulting in high levels of suspended solids due to anaerobic digestion. It is necessary to separate these phases using, e.g., pressure-driven processes – microfiltration and ultrafiltration [148]. Direct separation of solid and liquid phases can be performed in anaerobic membrane bioreactors, where the anaerobic process is followed by membrane separation (MF/UF) [149]. The digestate from the manure can be fractionated using a nanofiltration membrane, which very selectively separates phosphate ions from soluble nitrogen, phosphate recovery can reach more than 95% [150]. Electrodialysis reversal can also be utilized to separate phosphates from the digestate solution; although, the deposition of proteins and humic substances on the membrane results in fouling of the membranes [151]. The ManureEcoMine pilot process integrated thermophilic anaerobic co-digestion with ammonia removal (by producing ammonium sulfate using sulfuric acid) and precipitation of struvite in permeate after ultrafiltration. Thus, it was possible to process pig manure and plant residues while producing nitrogen and phosphorus fertilizers [152].

The combination of wet oxidation and precipitation of struvite was presented as effective process for phosphorus recovery [153]. A hybrid separation process consisting of wet oxidation and nanofiltration has resulted in the separation of more than 50% of phosphorus from sewage sludge. However, attention should be paid to the presence of other ions that affect nanofiltration separation and may reduce the efficiency of the process [46]. The pH of the solution is critical, phosphorus in the form of anions in an acidic environment (after the previous step of acidic leaching) can penetrate through nanofiltration membranes, where positive metal ions are retained [154]. Nanofiltration separation at low pH effectively retains multivalent metal cations, including iron, aluminum, and chromium by positively charged membrane surface because of ion size and electrostatic repulsion. It is caused by a stronger positive charge of the membrane under these conditions, which causes the repulsion of cations [155]. Other membrane methods, including electrodialysis, can be used for the selective separation of phosphorus species (mainly H_2_PO_4_ and H_3_PO_4_) from heavy metal ions present in sewage sludge ash [156]. Cation exchange membranes are also useful for separating unwanted metal ions present in sewage sludge (digested and incinerated) after prior acidic leaching [157]. It was found that the presence of solids and organic content negatively influenced the transport of cations through the membrane. The most interesting results were obtained for SSA because of reduced concentrations of these components. Utilization of membrane methods allows for P recovery for non-fertilizing applications. Although the main issues of their application are high material, and operating costs as well as the migration of heavy metals ions to the product. In spite of this research in the field of unwanted ions separation or improving the selectivity of the utilized technologies is recommended.

##### 3.2.3.2. Sorption

Phosphorus recovery is also possible through sorption in deposits characterized by an affinity for phosphorus forms present in the waste. In this case, the possibility of phosphorus recovery depends on many factors, including pH, affinity, phosphorus concentration in the waste, contact time (sorption kinetics), and sorbent capacity.

In a 2021 article, Li et al. (2021) achieved a phosphorus recovery of 86% using a modified natural raw material, diatomite. Due to the safety of its use, compared to acid leaching, it can be a method that allows relatively high efficiency with low financial outlays [158]. The utilization of adsorbents has become the subject of research and many research works in recent years [159,160]. Thermally treated organic materials seem to be the optimal solution for the adsorption of phosphorus compounds from sewage waste. *Posidonia oceanica* waste that has been thermally treated (500°C, 1 h) has been identified as the optimal adsorbent for the recovery of phosphorus with a capacity of 179.1 mg/g [161]. The authors evaluated the phosphorus extraction methods using sulfuric and nitric acid, where the use of H_2_SO_4_ under the influence of temperature allowed for a recovery of 93.2%. The trials in plants evaluated the effectiveness of the product obtained. The adsorbent was used as a fertilizer additive, which resulted in an increase in the germination rate to 111.14%, however, the utilization of material obtained from dewatered anaerobic sludge showed a negative effect on plant development. The authors presume that this may be related to harmful contaminants that have also been adsorbed from the source material. Research should be carried out to determine in detail which compounds from the dewatered anaerobic sludge cause a toxic effect on plants or to determine the absorption method, to limit the content of harmful elements. The process of thermal treatment/preparation of adsorbents for phosphorus recovery was also evaluated by Chen et al., (2018), who used bentonite in their research. Compared to biological waste, a wide temperature distribution was utilized here from 100 to 1000°C for 2 hours was utilized here. The adsorbent prepared at 800°C had a higher sorption capacity (6.94 mg/L) and was able to recover up to 94% of the phosphorus from the artificial environment. Unfortunately, the discussed study has only been performed on artificially produced phosphorus solutions without evaluation in the use of industrial waste. Although structural studies indicate that due to its rich crystal structure and stability to pH changes, it has such a high phosphorus binding capacity. The evaluation requires an attempt with real phosphorus-rich waste, as well as a subsequent evaluation of the release or extraction of adsorbed compounds.

Membrane technologies are still being developed, and technological improvements are necessary to determine their clear usability. Due to their high efficiency and the possibility of phosphorus separation in the liquid phase, they are a promising method, but the specificity of separation with respect to toxic metals must be increased and membrane clogging must be prevented (by prior hydrolysis or selection of raw materials).

#### 3.2.4. Thermal treatment

Animal manure can be treated hydrothermally at elevated temperatures and under high pressure. It is a thermochemical process that decomposes carbonaceous materials, the method takes advantage of the property that water under supercritical conditions has unique characteristics. As a result of the transition to a supercritical state, H bonds begin to degrade, which causes a decrease in the dielectric constant, and thus allows the dissolution of organic compounds while maintaining the possibility of dissolving the salt [162]. Depending on the temperature used, thermal hydrolysis (up to 170°C) or hydrothermal carbonization (up to 350°C) can be carried out. In both cases, manure can be applied without prior dehydration and changes in the liquid water reaction environment [117]. Pathogens are reduced during hydrothermal treatment, and phosphorus is transferred to the solid phase [52]. Municipal biological waste can also be subjected to this process, where it has been shown that higher efficiency can be obtained by increasing the parameters of the process. Recovery efficiency was >98% for 250°C, 90 min in the form of hydrochar. The extraction of other valuable compounds to the liquid and solid phase has been demonstrated, showing the closed cycle of a given method in fertilizer applications [163]. However, a direct evaluation of the application of these compounds to plant growth is required to determine the possible toxicity, which can occur due to the utilization of different sources of biological material.

The utilization of hydrothermal treatment with anaerobic digestion has been verified in terms of biogas production and phosphorus recovery both for biological waste (manure), and sewage sludge. It has been stated that the use of combined techniques is more efficient than the application of single processes. The biogas production was significantly improved by exposure to 125°C compared to other treatment parameters (700 mL/g VS). The thermal pretreatment seems to improve the crystallinity of the recovered phosphorus in the solid phase. It is assumed that amorphous calcium phosphate converts to hydroxyapatite and phytate also undergoes reduction through the degradation of organic forms due to the effect of temperature treatment. This improves the overall recovery of P from the solid phase through the combined processes. In the case of the liquid phase, the thermal process at 125°C improved the total P content by about 1.7% for sewage sludge, and then after 63 days of the anaerobic digestion, the improvement reached 4.9%. Although, the treatment of biological waste (manure) has been shown to deteriorate by 3.5% and 0.9% in these processes, respectively. This indicates the essence of the appraisal of particular combinations of technologies with regard to the influence on a certain waste [164].

Hydrothermal treatment with steam gasification can be used to recover phosphorus from sewage sludge. The authors Feng et al. (2018) propose such a solution by optimizing it in terms of the applied hydrochar preparation temperature, where it was indicated that the highest recovery rate in combined processes is obtained for 200°C. However, an evaluation of the temperature influence on changes in phosphorus form was performed, where for temperatures up to 240°C there was a partial change of organic phosphorus to inorganic, and at the 260°C phosphorus oxides were emitted. It was indicated that increasing pH and adding CaO improved the efficiency of the transformation of Al, Fe, and Mn forms into the phosphates bound to the Ca^2+^ ion. Those forms have a better potential for industrial application. This trend was confirmed in the publication by Y. Shi et al., (2019), where the process allowed a high recovery of phosphorus in the forms of calcium compounds that can be used for agricultural purposes. The method of phosphorus transfer into the liquid phase was also carried out by the utilization of HCl as an additive to the production process. It was determined that, thanks to the transfer of phosphorus to the liquid phase, a crystallization process (pH 7.52) could be utilized to obtain a highly pure struvite (90.41%). The post-gasification waste evaluation was carried out by Gorazda et al. (2018), who obtained large amounts of phosphorus by extraction using phosphoric and nitric acids. The method allows reducing the waste generated in the gasification process developed in the work by Feng et al., (2018). The recovered phosphate does not contain large amounts of harmful heavy metals and iron, which remain in the solid phase. Combining a method allows for a circular approach economy with minimal waste production and loss of desired compounds.

The publication by Wang et al. (2019) evaluated the issues of heavy metal migration due to the hydrothermal carbonization process [182]. It was indicated that an increase in the process conditions for sewage sludge could lead to a higher amount of phosphorus compounds in the hydrochar. The process parameters of 300°C and 180 min showed a greater degree of conversion into inorganic phosphorus forms, although non-apatite forms decreased their presence in the solid phase, as well as orthophosphate forms in the liquid phase. Analysis of composition changes in terms of heavy metal content showed a decrease in bioavailable fractions but an increase in stable ones. This may be the reason for inhibiting plant development despite the rich elemental composition. It is recommended to evaluate the effect of hydrochar on plant growth or utilize it for other industrial applications instead.

The pyrolysis method was evaluated in the paper by Steckenmesser et al. (2017), the low- and high-temperature pyrolysis was evaluated [183]. The process consists of the manipulation of temperature to decompose chemical bonds. It consists of two main steps, the first one involves primary pyrolysis releasing volatiles, and forming char. The produced heat is then transferred between the individual elements of the cooler part of the treated material. This indirectly causes the volatiles to condense and create tar. Autocatalytic secondary pyrolysis occurs at the same time as primary pyrolysis, which causes further decomposition of the material and products of primary pyrolysis. This is mainly due to the fact that the pyrolysis process, based on a given material component, may be exothermic (lignin) or endothermic (cellulose), which explains the simultaneous effect of two different processes [165]. It has been shown that the application of low-temperature pyrolysis from 400°C to 500°C allows the high bioavailability of phosphorus forms for plants. These results from the polymerization into more condensed phosphates. This tendency of utilizing a low temperature was confirmed in the work of Meng et al. (2021), wherein the co-combustion process (<600°C) the degree of non-digestible forms of Fe, Al phosphates to Ca forms was elevated [166]. However, this method can only be used on sewage sludge with a low heavy metal content and without the chemically recovered phosphorus. In the case of material with high toxic compounds or where P was removed by biological treatment, the authors suggest pyrolysis at 950°C in the presence of Na_2_SO_4_. This allows the obtaining of calcium-sodium-phosphates, which are better absorbed by plants than the forms of Fe, Al, and Ca.

Incineration as one of the thermal methods utilized for the recovery of phosphorus from sewage sludge indicates the best balance in terms of economic aspect and efficiency. Apart from recovering P from waste, it generates thermal energy, which can be reused for other industrial applications. The process itself consists of four main parts: sludge pretreatment, combustion, energy recovery, and cleaning systems. Although, the key operation is the combustion (up to 950°C), where the organic matter is completely burned and converted to gas, and the remaining inorganic phase, including the recovered phosphorus, is transferred to the solid phase as ash. The resulting hot gases are then directed to the energy recovery system and then converted into electricity or heat [167]. Efficiency can be up to 80% [168]. However, there is a problem with poor application possibilities of the obtained material due to non-bioavailable forms of phosphorus. This issue was evaluated in the article by Xu et al., (2021) where the addition of MgCl_2_ and CaCl_2_ was proposed in order to improve the degree of conversion of phosphorus from non-apatite inorganic to phosphorus apatite. These chlorides have been shown to react with AlPO_4_, which plants do not assimilate. The transformation can occur at a temperature of 500–600°C for Ca ions and 700–750°C for Mg ions. Both products are highly bioavailable to plants, extending the applications of incineration.

Almost 90% of polyP (mainly nucleic acids and phospholipids) can be released from activated sludge cells by simple heating at 70–90°C. This technology has been implemented on a pilot (Heatphos) and full-scale scale in Japan with an efficiency of 10 kgP/day [169]. The mechanism of this method is not fully explained. Although, by heat treatment, it is possible to transport polyP from the cytoplasm of microorganisms [170]. This is most likely due to the disturbance of the walls of bacteria from the sewage sludge. During the thermochemical process, the addition of a chlorine donor (such as alkaline earth metal chlorides) to SS or SSA in the presence of high temperature (750–1050°C) releases volatile heavy metal chlorides [171]. At the same time, phosphorus is converted into mineral phosphates, which are readily available to plants.

Phosphorus recovery technologies from biowaste and wastewater sludge (Table 4) allow effective phosphorus separation. Consideration should also be given to the parameters of the waste and sewage sludge itself (composition, quantity) and to local possibilities of its management [37]. The major technological challenge remains the removal of unwanted heavy metals. Additional separation processes such as ion exchange adsorption or membrane processes allow the final product to be purified from other components such as heavy metal ions.

**Table 4.** Effectiveness of selected phosphorus recovery technologies from biowaste and sewage sludge

Technology	Feedstock	Efficiency/ Recovery rate	Comments	References
Co-composting	paddy straw with cattle manure, farm yard manure and poultry manure	49–57%	addition of a consortium of phytate mineralizing fungi organic fertilizer with high P availability	[172]
Extraction – Hedley fractionation	animal manure	>96%	sequential extraction: deionized water, 0.5 M NaHCO_3_, 0.1 M NaOH, and 1.0 M HCl	[173]
Acid extraction	ashes of poultry litter meat and bone meal ashes	90%	H_2_SO_4_ extraction was more effective compared to HNO_3_. phosphorus recovery was linearly dependent on the pH below pH ~ 4	[191]
Hydrothermal treatment in the presence of acids	swine manure	94%	maximum efficiency for extraction with H_2_SO_4_ at 170°C	[52]
Acid extraction	pig manure solids	87%	balanced N:P ratio for crop production	[130]
Pyrolysis + acid extraction	pig manure	90%	nitrogen loss during pyrolysis requires the addition of N to the final fertilizer	[174]
Heatphos	SS	87%	simple heating at 70°C for 1 h polyphosphate releasing and precipitating after CaCl_2_ addition	[87]
Thermochemical treatment	SS	>95%	Cl donor: MgCl_2_ or CaCl_2_ removal of heavy metals	[175]
Hybrid process of low-pressure wet oxidation and nanofiltration	SS	54%	reduced emission of greenhouse gases removal of heavy metals	[46]
Supercritical water oxidation	SS	90%	extraction of P in caustic recovery and reuse of caustic	[176]
Supercritical water process	SS	85%	possible reuse of extractants	[177]
Wet chemical extraction method	SSA	25%-40% for chelating agents >90% for acids	extractants: inorganic acids (H_2_SO_4_, HNO_3_) organic acids (oxalic acid and citric acid), and chelating agents (EDTA and EDTMP) organic acids extract more trace elements compared to inorganic acids H_2_SO_4_ selected as optimal extractant	[142]
Wet chemical extraction method	SSA	>95%	extractants: H_2_SO_4_ and oxalic acid products: struvite H_2_SO_4_ extract and aluminum and iron hydroxyphosphates from the H_2_C_2_O_4_ extract	[145]
Wet chemical extraction method	SSA	>86%	extraction with HNO_3_	[178]
Four-step process	SSA	79.7%	acid extraction, alkali precipitation, cation exchange resin adsorption, struvite crystallization	[179]
Electrodialysis	SSA	59%	separation of suspension SA with H_2_SO_4_ 14 days	[180]
Donnan process	DSS	>60%	extraction with 25% H_2_SO_4_ a Donnan membrane with cation exchange membrane – removal of Al, Ca, Fe	[157]
Adsorption *(Posidonia oceanica*)	dewatered anaerobic sludge	76%-98.3%	extraction with H_2_SO_4_ and thermal treatment possible absorption of harmful compounds	[161]
Hydrothermal carbonization	biogenic municipal waste	91.06%-98.92%	hydrochar (high P and N content), liquid phase (high Na and K content) can be used for fertilizing purposes P recovery increases with temperature and duration of the process	[163]
Hydrothermal treatment with steam gasification	SS	84.92%	best results for hydrochar prepared at 200°C with the addition of CaO most of the phosphorus compounds related to Al, Fe, Mn have been transformed into forms tied to Ca	[189]
Incineration	SS	80%	low cost	[168]
Hydrothermal treatment	dewatered sewage sludge	98.37%	increasing the temperature causes an increase in P-Ca forms the addition of HCl to the process causes the transport of phosphorus to the liquid phase	[188]
Chemical extraction	solid gasification residue	73–82%	extraction with nitric acid and phosphoric acid	[181]
Bacterial treatment (*Acidithiobacillus thiooxidans)*	SS	57%	long time – 27 days sulfur supplementation increases efficiency	[184]
Sorption on modifying diatomite	SS	86%	depends on pH, contact time possibility of regeneration of the absorbent	[158]

DSS – digested sewage sludge, SS – sewage sludge, SSA – sewage sludge ashes

## 4. Guidelines for a practical approach

The literature has analyzed phosphorus recovery methods from the most popular high phosphorus waste. Recovery systems used in water conditioning facilities, according to Table 3, reach a maximum efficiency of 50% efficiency; in other cases, it is an average of 20%. These values are obtained mainly through precipitation or crystallization, which allows the receiving of the fertilizer material in the form of struvite, ready for agricultural application [81,86]. The optimal treatment method cannot be unequivocally determined on the collected data. For Type I of the P source, none of the technologies shown seems to be the most effective solution. On the basis of the ANPHOS technology information, it can be assumed to be the optimal solution. The main reason is a 90% recovery rate when utilized in wastewater from the potato industry. ANPHOS technology introduces a batch process, so introducing pretreatment or expanding a biological method, which requires a lot of time for microorganisms to work, seems easier than in other solutions [71]. For type II P waste, AirPrex was only utilized to provide up to 50% recovery. Many intermediate processes demonstrate the possibility of introducing intermediate stages of sewage preparation for phosphorus recovery [186]. Type III of P waste allows for constant 50% phosphorus recovery when using Gifhorn technology with 90% removal efficiencies from influent [37,149]. It is proposed to introduce chemical hydrolysis as a pretreatment of the wastewater process, which can increase the efficiency of the recovery methods used.

The treatment of various types of wastewater sludge based on the data in Table 4, can be assumed that thermal methods are the optimal solution. The process efficiency and reduction bioavailability of the heavy metals without the need to separate them from the fertilizer medium allow their direct application. Among the thermal methods, the most favorable seems to be incineration, which offers a high recovery rate while maintaining relatively low operating costs [168]. However, the main disadvantage of this method is the small number of possible applications due to the non-bioavailable forms of P obtained through its utilization. This problem has been solved by adding CaCl_2_ and MgCl_2_, which allow one to change the forms of phosphorus to those available for plants [187]. This indicates the potential of this technology in terms of fertilizer extraction. Hydrothermal treatment with gasification allows recovery efficiency of up to 84.92% (200°C) [188]. The process itself results in the production of two phases containing phosphorus. Using HCl, phosphorus transport from the solid phase to the liquid phase is possible. This property can be used for crystallization at pH 7.52 to obtain struvite with a purity of 90.41% [188]. The hydrothermal treatment process itself is highly modifiable in changing the efficiency of the recovery rate and its forms by changing the pH or adding CaO [189]. The process of gasification mentioned above produces a large amount of waste, which, through the use of nitric and phosphoric acid, allows for additional phosphorus recovery. Those publications indicate the possibility of providing a low-waste method consistent with the circular economy approach. The potential of combining removal methods was also demonstrated in the article by Zuo et al. (2016), where the possibility of using the HA-A/A-MCO (Hydrolysis-Acidogenosis-Anaerobic/Anoxic-Multiple Continuous Oxic Tank) process was evaluated with removal of up to 95.2% P [185]. As in the case of the other methods, the utilization of adsorbents for the recovery of P can be the optimal solution in terms of reducing energy costs. In times of a energy production crisis, this increases its potential usage, where the adsorbed material can be used as fertilizing additives or as an extraction material for other applications. The main conclusion of the researchers is the need to introduce pretreatment for the use of adsorbents to increase the capacity and selectivity of the material for the recovery of phosphorus. Pretreatment, which is commonly used, is the thermal treatment used to change the structure of inorganic compounds or obtain biochars from biological materials. Posidonia oceanica exposed to 500°C for 1 h has a high adsorption capacity and selectivity for P recovery [161]. This indicates the potential for utilization of other biological waste for such applications. However, inorganic materials were also evaluated, in this case, bentonite, which was subjected to thermal treatment at 800°C for 2 hours [190]. This pretreatment allowed it to remove up to 94% of phosphorus. Thermal treatment has been described to cause crystal structure of the adsorbents and increase stability to pH changes. For the extraction of P, sulfuric acid was used, resulting in the removal of 93.2% P from the adsorbent, which is a highly satisfactory method [161]. The described method can be a cheap way to separate P using biological or inorganic waste. This will reduce environmental pollution from those types of waste and increase the amount of phosphorus that is possible to recover. It is essential to evaluate the composition of the adsorbed material for the presence of toxic compounds that can also be adsorbed from highly contaminated high-phosphorus waste. Based on available research, the method seems to be optimal to utilize on waste with a low concentration of heavy metals.

**Figure 9.**
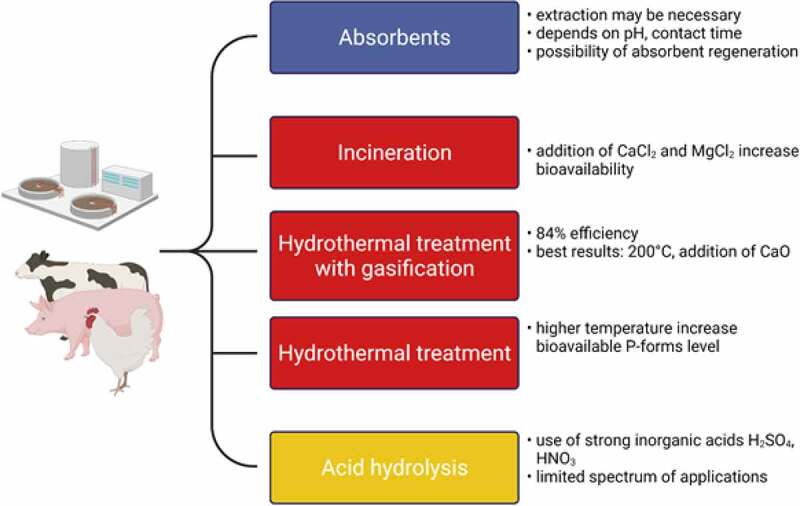
Overview of potential methods to recover phosphorus, blue – physical, red – thermal, yellow – chemical.

Similar conclusions could be drawn in the case of biological waste from breeding and households. Thermal methods, particularly hydrothermal treatment, are the most optimal solutions for the recovery of P and other micro- and macroelements. It allows one to obtain fertilizer at relatively low temperatures (250–350°C) with an efficiency of 98% [163]. Acid hydrolysis seems to be a promising solution as well. The recovery rate was evaluated using strong inorganic acids H_2_SO_4_, HNO_3_ and was evaluated that it can be up to 87–90% [130]. The method itself is simple and cheap, although it requires optimization for each extraction medium and recovery rate on pH. The hydrolyzates obtained have fertilizing potential, mainly due to the recovery of the remaining nutrients, but the spectrum of other applications seems limited. The Hedley fractionation method indicates a recovery rate >96% by utilizing sequential extraction by deionized water, 0.5 M NaHCO_3_, 0.1 M NaOH, and 1.0 M HCl. A major advantage of chemical treatment is utilizing any organic waste, in the form of organic fertilizer. The process itself can be modified by applying thermal methods such as pyrolysis to provide different forms of products without decreasing the P content. The optimal parameters for the recovery of P from waste have been shown in Figure 9.

## 5. Discussions and conclusions

Researchers still investigate studies associated with the possibilities of P removal from wastewater and biological waste, leaving many gaps and areas for further exploration. Phosphorus recovery methods still seem incomplete and have the potential to introduce pretreatment of the extraction material or modification of available technologies. The low phosphorus recovery rate to a maximum of 50% with such large amounts of waste material indicates the need to conduct more research in this area. It is recommended to carry out studies on the P recovery by involving chemical hydrolysis and thermal treatment to remove excessive heavy metals and change the forms of phosphorus to more suitable for further application or by providing biological treatment in the batch process.

Based on papers on wastewater treatment facilities, it is recommended to carry out comprehensive trials in the usefulness of the field of the obtained fertilizer on plant growth. Analysis in terms of heavy metal composition, chemical potential, or microbiological compounds that can adversely affect plant development seems to be the most critical issue with respect to obtained products. The multitude of materials and types of municipal and industrial waste requires a verification of the suitability of the materials. Several studies indicated that, depending on the waste material from which the phosphorus was recovered, fertilizer can have different consequences on plant growth. Despite a good composition of micro and macroelements, the products negatively affected plant growth, which was caused by a predominantly heavy metal content or harmful microorganisms.

Despite its considerable application potential, adsorption methods require expanding research in the field of the elemental and microbiological composition of the recovered phosphorus. It has been concluded that the source of phosphorus influences the final efficiency of the adsorbent as a fertilizing additive, and therefore, a comprehensive evaluation of the final product based on the waste source is recommended. Research points out that the mentioned method is a cheap alternative to the commonly used techniques, such as thermal and chemical, mainly due to the simplicity and variety of applications on different waste materials. The possibility of utilizing waste materials as adsorbents and in combination with biological phosphorus recovery methods seems to be a noteworthy research direction.

It is recommended to conduct studies on the use of combined methods in the concept of circular economy, as was the case with hydrothermal treatment with gasification [189], indicating a high potential for the recovery of phosphorus and waste-free production. Chemical treatment combined with the utilization of thermal methods seems to be the future of phosphorus recovery, which was proven by the papers carried out on hydrothermal treatment in the presence of acids and pyrolysis with acid extraction. These technologies resulted in higher recovery efficiency higher than that of a single application of the demonstrated techniques.

The critical issue is the assessment of the obtained phosphorus forms and their bioavailability dependent on the process conditions applied. The key aspects are to research the effects of additives such as CaO, MgCl_2_, CaCl_2_ to determine the optimal solution in this area. This will allow for the extension of utilization of recovered phosphorus, whereas the forms of phosphorus vary considerably in bioavailability to plants.

The use of mixed methods for the treatment of sewage sludge and biowaste indicated better results for phosphorus recovery. It has been shown that the application of modifications and additives to separate technologies is conducive to both increasing efficiency and obtaining bioavailable forms of phosphorus for agricultural use. Research on the utilization of adsorbents also specifies a way to acquire phosphorus while minimizing costs. Although there has been a concern about adsorbing too many toxic compounds, the potential benefit for fertilizing purposes points out the need to expand research in this area.
